# Genetic association of *OPR* genes with resistance to Hessian fly in hexaploid wheat

**DOI:** 10.1186/1471-2164-14-369

**Published:** 2013-06-01

**Authors:** Chor Tee Tan, Brett F Carver, Ming-Shun Chen, Yong-Qiang Gu, Liuling Yan

**Affiliations:** 1 Department of Plant and Soil Sciences, Oklahoma State University, Stillwater, OK 74078, USA; 2 Department of Entomology, Kansas State University, Manhattan, KS 66506, USA; 3 United States Department of Agriculture, Agricultural Research Service, Genomics and Gene Discovery Research Unit, Albany, CA 94710, USA

**Keywords:** Hessian fly resistance, Insect resistance pathway, *lipoxygenase* (LOX), *12-oxophytodienoic acid reductase* (OPR), Quantitative trait loci (QTL), Wheat

## Abstract

**Background:**

Hessian fly (*Mayetiola destructor*) is one of the most destructive pests of wheat. The genes encoding 12-oxo-phytodienoic acid reductase (OPR) and lipoxygenase (LOX) play critical roles in insect resistance pathways in higher plants, but little is known about genes controlling resistance to Hessian fly in wheat.

**Results:**

In this study, 154 F_6:8_ recombinant inbred lines (RILs) generated from a cross between two cultivars, ‘Jagger’ and ‘2174’ of hexaploid wheat (2n = 6 × =42; AABBDD), were used to map genes associated with resistance to Hessian fly. Two QTLs were identified. The first one was a major QTL on chromosome 1A (*QHf.osu-1A*), which explained 70% of the total phenotypic variation. The resistant allele at this locus in cultivar 2174 could be orthologous to one or more of the previously mapped resistance genes (*H9*, *H10*, *H11*, *H16*, and *H17*) in tetraploid wheat. The second QTL was a minor QTL on chromosome 2A (*QHf.osu-2A*), which accounted for 18% of the total phenotypic variation. The resistant allele at this locus in 2174 is collinear to an *Yr17*-containing-fragment translocated from chromosome 2N of *Triticum ventricosum* (2n = 4 × =28; DDNN) in Jagger. Genetic mapping results showed that two *OPR* genes, *TaOPR1-A* and *TaOPR2-A*, were tightly associated with *QHf.osu-1A* and *QHf.osu-2A,* respectively. Another *OPR* gene and three *LOX* genes were mapped but not associated with Hessian fly resistance in the segregating population.

**Conclusions:**

This study has located two major QTLs/genes in bread wheat that can be directly used in wheat breeding programs and has also provided insights for the genetic association and disassociation of Hessian fly resistance with *OPR* and *LOX* genes in wheat.

## Background

Hessian fly [Hf, *Mayetiola destructor* (Say)] is one of the most destructive pests of hexaploid wheat (*T. aestivum* L., AABBDD, 2n = 6 × =42) in the United States and worldwide [1]. Hessian fly larvae live between leaf-sheaths at seedling stage in fall, inhibit wheat growth irreversibly, and the infested plants lose vigor and die after larvae become pupae. Hessian fly larvae also attack stalks in spring, and the attacked plants may break easily before harvest. Deployment of natural and genetic resistance in locally adapted wheat cultivars is the most effective, economical, and environmentally safe method to control this devastating insect.

At least 33 Hessian fly resistance genes have been identified, designated as *H1* to *H32* and *Hdic*[2]*.* Among these Hessian fly resistance genes, however, only 8 genes (*H1-H5*, *H7*, *H8*, and *H12*) were identified in hexaploid wheat [3-8]. The remaining 25 genes were identified in distant and close relatives of hexaploid wheat. There are 15 genes (*H6*, *H9*–*H11*, *H14*–*H20*, *H28*, *H29*, *H31*, and *Hdic*) identified in tetraploid wheat species *Triticum turgidum* subsp. *durum* (AABB, 2n = 4 × =28) [9-19], and 6 of them (*H6*, *H9-H11*, *H31*, and *Hdic*) have been introgressed from tetraploid to hexaploid wheat [7,18-20]. Other genes originating in wild diploid wheat or other relatives and introgressed to hexaploid wheat include six genes (*H13*, *H22*, *H23*, *H24*, *H26*, and *H32*) from *Aegilops tauschii* (DD, 2n = 2 × =14) [21-25], two genes (*H21* and *H25*) from *Secale cerale* L. (RR, 2n = 2 × =14) [26,27], one gene (*H27*) from *Aegilops ventricosa* (D^v^D^v^M^v^M^v^, 2n = 4 × =28, [28]), and one gene (*H30*) from *Aegilops triuncialis* (CCUU, 2n = 4 × =28) [29]. These genes provide important resistance sources but are problematic in variety development programs when they are associated with alien linkage drag.

Simple sequence repeat (SSR) or microsatellite markers were used to map Hessian fly resistance genes in previous studies. Seven genes (*H5*, *H9*, *H10*, *H11*, *H16*, *H17*, and *Hdic*) were mapped on chromosome 1A, and these genes may comprise a cluster (or family) of Hessian fly resistance genes in the distal gene-rich region of wheat chromosome 1AS [19,20,30,31]. The remaining mapped genes include eight genes (*H3*, *H6*, *H12*, *H14*, *H15*, *H19*, *H28*, and *H29*) on chromosome 5A [10,17,32,33], three genes (*H24*, *H26*, and *H32*) on chromosome 3D [23-25], three genes (*H18*, *H20* and *H21*) on chromosome 2B [16,26,29], two genes (*H13* and *H23*) on chromosome 6D [23,34], *H7* on chromosome 5D [35], *H22* on chromosome 1D [22], *H25* on chromosome 6B [27], *H31* on chromosome 5B [18], and *H27* on chromosome 4D [28]. Current understanding of SSR markers is that they can be effectively applied to marker-assisted selection (MAS) due to their relative simplicity. However, the ‘repeat’ feature of an SSR marker results in multiple and inconsistent locations of the same marker among divergent wheat cultivars [36]. Only a gene marker that is developed for the specific functional polymorphism of a gene responsible for a given trait can provide the ultimate resolution needed for selection of the trait [37].

The best strategy to find the regulatory sites of a gene is to clone the gene/QTL. The cloned gene is then used to develop perfect gene markers for use in conventional breeding programs or to manipulate transgenic wheat. To date, 14 genes have been cloned from wheat using the positional cloning strategy [38,39], but no gene has been cloned for resistance to Hessian fly. To clone a gene from hexaploid wheat using the map-based cloning approach remains a challenge because of the complexity imparted by three homoeologous genomes, the large genome size (17 Gb), high content of repetitive sequences (>80%), and the low polymorphism rate [40-43]. In recent studies, genetic association is also use to identify functional genes for important traits in wheat [44].

Recent progress in the application of high-throughput sequencing technologies and development of sophisticated genomic mapping tools has accelerated identification of agriculturally important genes in wheat. Numerous wheat ESTs assigned to wheat deletion bins have facilitated the physical mapping of a gene [45,46]. The availability of genomic sequences from the model species rice [45] and *Brachypodium distachyon*[47], and synteny conservation in certain genomic regions between these species and wheat [48,49] have allowed more precise determination of the physical distance between two markers flanking the target gene by a comparative genomics analysis. Newly available wheat genomic sequences, though at low coverage (http://www.cerealsdb.uk.net/), are useful for designing primers specific to a homoeologous gene for genomic mapping. With the integration of these techniques and tools, mutants of several genes including *VRN-A1*[50], *VRN-D3*[50], *PPD-D1*[50], *Lr34-D*[51], *Yr17*[52], and *Pm3*[37] have been identified in cultivated bread wheat. These genes each were initially mapped under the peak of a QTL for a relevant trait and their mutants were eventually confirmed. In this study, we aimed to determine if any genes known to confer insect resistance in plants are associated with a QTL for resistance in our mapping populations or in a genomic region where a QTL has been reported in published mapping populations.

Plants possess multiple defense mechanisms in response to mechanical damage due to insect attack. Jasmonic acid (JA) and its conjugates, jasmonates, play a central role in regulating defense responses of plants to insect herbivores [53]. In higher plants, JA is synthesized via the octadecanoid pathway consisting of several enzymatic steps. The early steps of this pathway occur in chloroplasts where linolenic acid is converted to 12-oxo-phytodienoic acid (OPDA) by means of three enzymes, lipoxygenase (LOX), allene oxide synthase (AOS), and allene oxide cylase [54-56]. OPDA is subsequently reduced in a cyclopentenone ring by a peroxisome-localized enzyme, 12-oxo-phytodienoic acid reductase 3 (OPR3). The reaction product then undergoes three cycles of oxidation in the peroxisome, generating JA [57-59].

In this study, we mapped 3 *OPR* genes and 3 *LOX* genes in a population of recombinant inbred lines (RILs) that was generated from a cross between two locally adapted winter cultivars, ‘Jagger’ and ‘2174’, and showed demonstrable segregation for resistance to Hessian fly. The implication in association and disassociation of the QTLs for resistance to Hessian fly with these *OPR* and *LOX* genes is discussed.

## Results

### QTLs mapped for wheat resistance to Hessian fly

**Figure 1:**
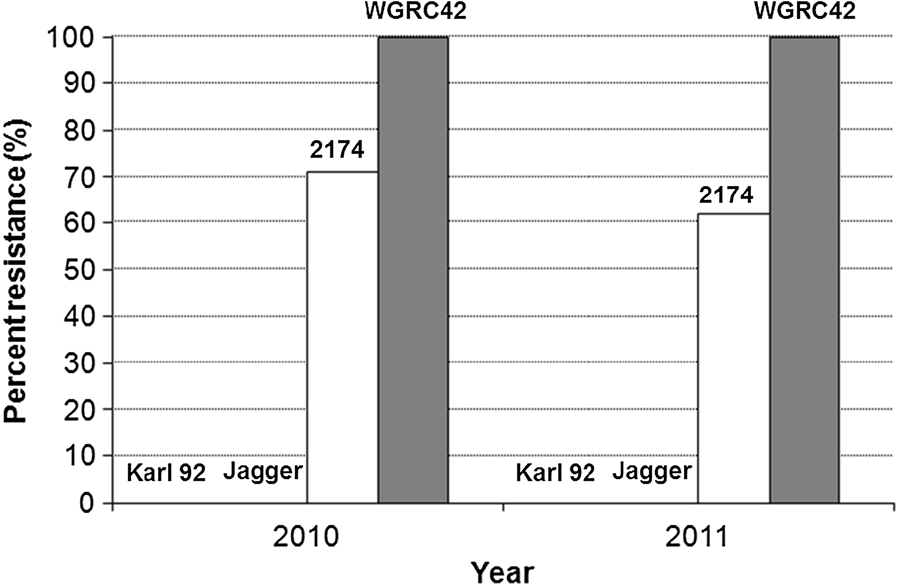
**Comparative analysis of Hessian fly resistance among hexaploid wheat cultivars.** Ratings were scored [67] for two-years for Jagger and 2174 used as the parental lines to generate RILs. Karl 92 and WGRC42 were used as susceptible and resistant controls, respectively.

Jagger was highly susceptible and 2174 was highly resistant to Hessian fly biotype GP when the two parental lines were tested with susceptible (Karl 92) and resistant (WGRC42) cultivars as controls (Figure 1). Hence variation in Hessian fly resistance between the two parental lines should facilitate mapping the resistance trait based on segregation in the available population of F_6:8_ RILs generated by crossing the parental cultivars.

**Figure 2:**
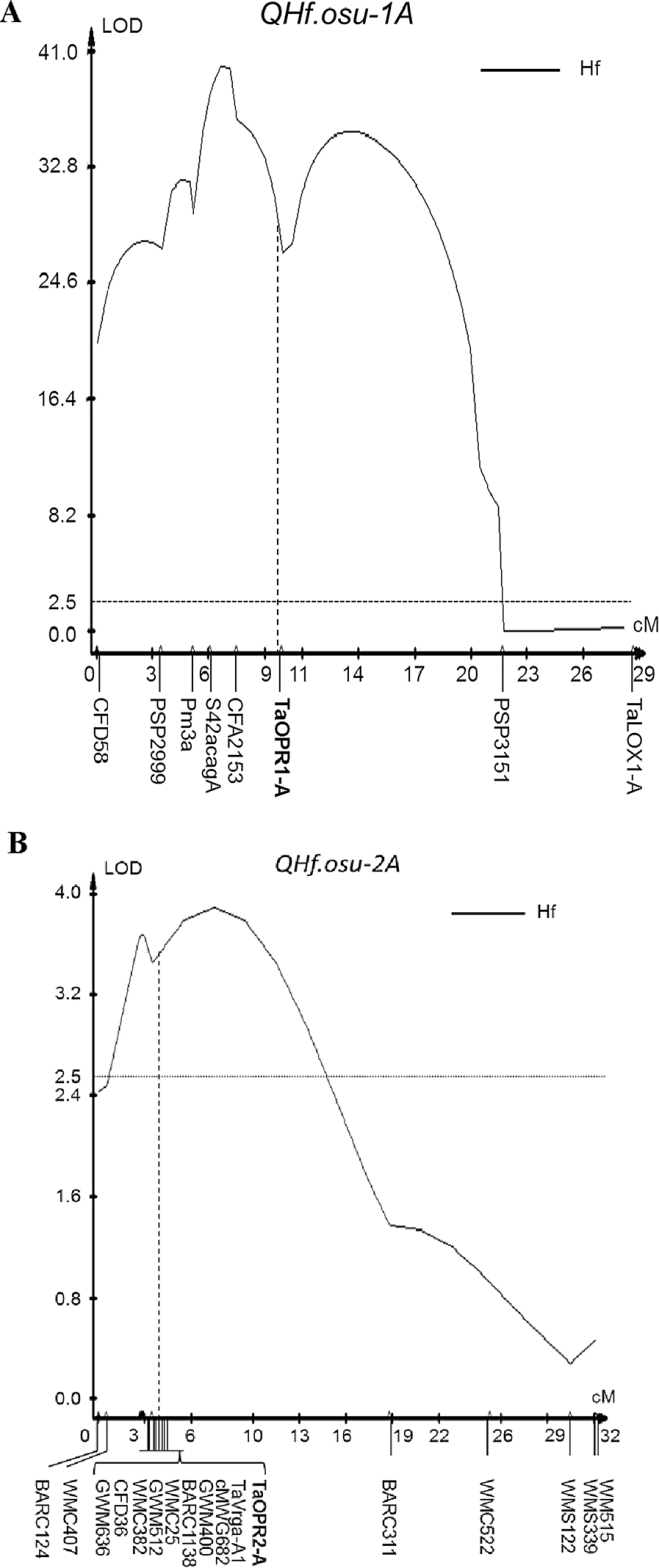
**Mapping of two QTLs for resistance to Hessian fly.** Response to Hessian fly for each of 154 RILs was scored in the Jagger × 2174-derived RIL population using the method previously described [67]. QTL analysis was performed with composite interval mapping (CIM) using WinQTLCart 2.5. The positions of marker loci are shown on the x-axis in centiMorgan (cM) distances. The horizontal dotted line indicates the logarithm of the odds (LOD) significance threshold of 2.5. The vertical dash line indicates the gene markers associated with the QTLs.

Using 154 RILs of the Jagger × 2174 population and genome-wide SSR markers [52], we mapped two QTLs linked with Hessian fly resistance. The first one was a major QTL on the distal end of chromosome 1AS (*QHf.osu-1A*) (Figure 2A), when the population was infested with the same biotype as the parental lines. *QHf.osu-1A* was mapped in tight linkage with the powdery mildew gene *Pm3a* previously identified [37]. The LOD value for *QHf.osu-1A* was 28.7, which explained 70% of the total phenotypic variation. Phenotypic data showed that those RILs which carried the Jagger allele had resistance at the level of 6%, whereas those RILs that carried the 2174 allele had resistance at the level of 59%. 2174 has a resistant allele for both Hessian fly and powdery mildew (*Pm3a*) genes.

The second QTL was a minor QTL on chromosome 2A (*QHf.osu-2A*) (Figure 2B). The peak of this QTL was associated with a group of several tightly linked SSR markers and the marker for *Yr17* that was translocated from chromosome 2N of *Triticum ventricosum* (2n = 4 × =28; DDNN) in Jagger [52,60]. The LOD value for *QHf.osu-2A* was 3.5 and this QTL accounted for 18% of the total phenotypic variation. Phenotypic data showed that those RILs which carried the Jagger allele had resistance at the level of 23.1%, whereas those RILs that carried the 2174 allele had resistance at the level of 51.9%. The resistant allele at this locus in 2174 is collinear to an *Yr17*-containing-fragment translocated from chromosome 2N in Jagger. Jagger attained the translocated chromosome 2N fragment conferring resistance to stripe rust [52]. If the translocated fragment in Jagger was introgressed into a breeding line or a cultivar such as 2174, the novel breeding line could lose approximately 60% of Hessian fly resistance conferred by the *OPR2-A* gene on chromosome 2A in 2174.

### Associations of QTLs for Hessian fly resistance with two *OPR* genes

**Figure 3:**
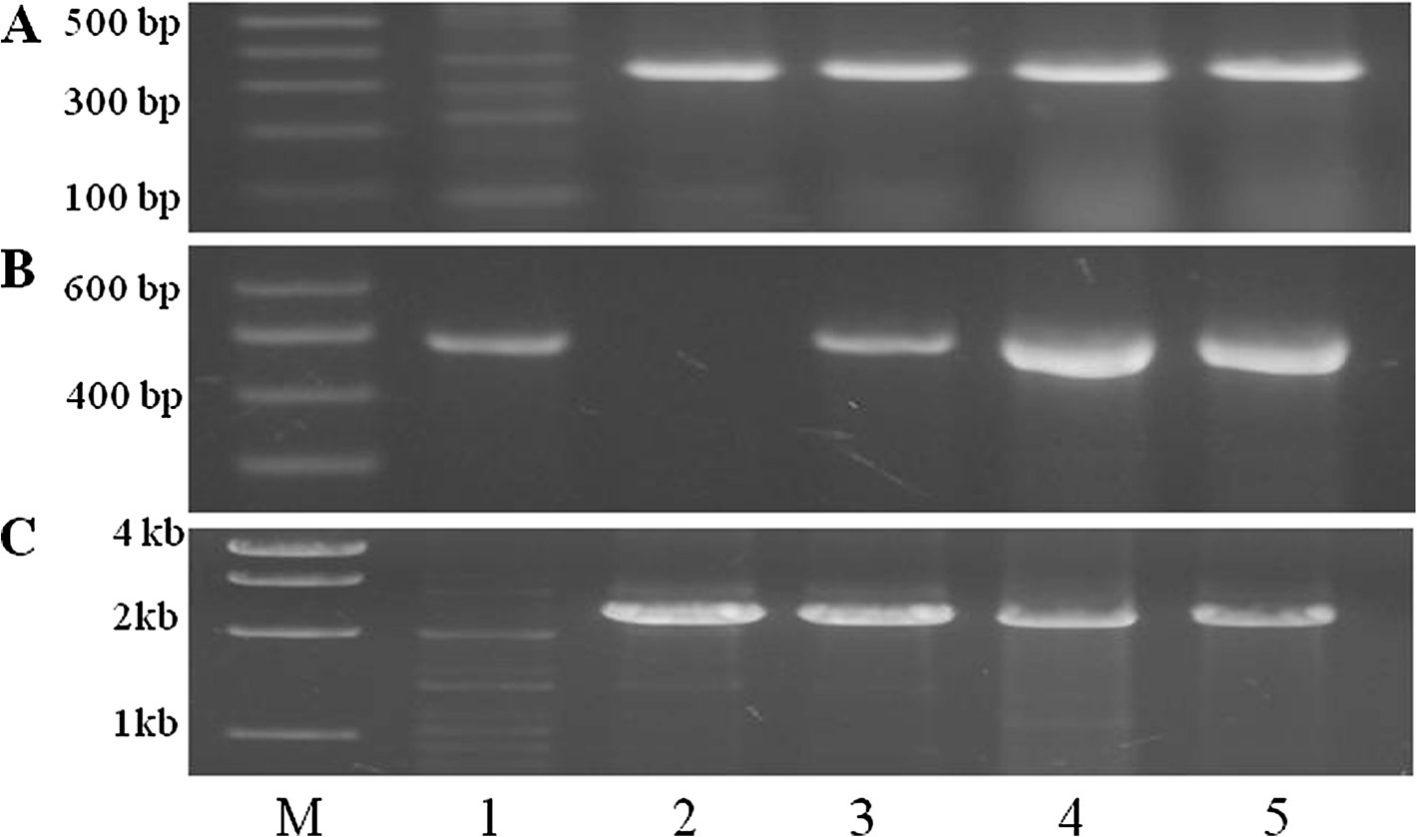
**Gene markers tested on N1AT1D, N1BT1D, and N1DT1B of CS nullisomic–tetrasomic lines.** PCR products amplified for *TaOPR1-A, TaLOX1-A,* and *TaOPR7-B*. Lanes: 1 N1AT1D, 2 N1BT1D, 3 N1DT1B, 4 Jagger, 5 2174. M Molecular marker. **A**) *TaOPR1-A* marker. PCR was performed using primers OPRC1-ABD-F2 and OPRC1-R8. **B**) *TaOPR7-B* marker. PCR was performed using primers OP1-C1F1 and OP1-R1. **C**) *TaLOX1-A* marker. PCR was performed using primers LOX-C5-F5 and LOX-C5-R6. Expected size of PCR products was indicated in Table 1.

The peak of *QHf.osu-1A* was centered with SSR marker *Xcfa2153* (Figure 2A), which was physically located in a deletion bin of 1AS-3 (FL 0.86), or the distal 14% of the short arm of chromosome 1A [61]. Wheat EST BE403717 encoding an OPR protein was also found present in this 1AS-3 FL 0.86 deletion bin. It is thus intuitive to hypothesize that this *OPR* gene may be a candidate gene for *QHf.osu-1A* if it is mapped under the peak of this QTL. The BE403717 sequence was used to blast against the wheat genomic DNA databases (http://www.cerealsdb.uk.net), and the wheat sequences retrieved from the databases were grouped based on sequence alignments of the homoeologous and paralogous *OPR* genes. Primers were designed for each group of these genes and tested for specificity to N1AT1D, N1BT1D, and N1DT1B of ‘Chinese Spring’ (CS) nullisomic–tetrasomic (NT) lines [62]. Primers OPRC1-ABD-F2 and OPRC1-R8 for one of the *OPR* genes were identified specific to chromosome 1A (Figure 3A).

**Figure 4:**
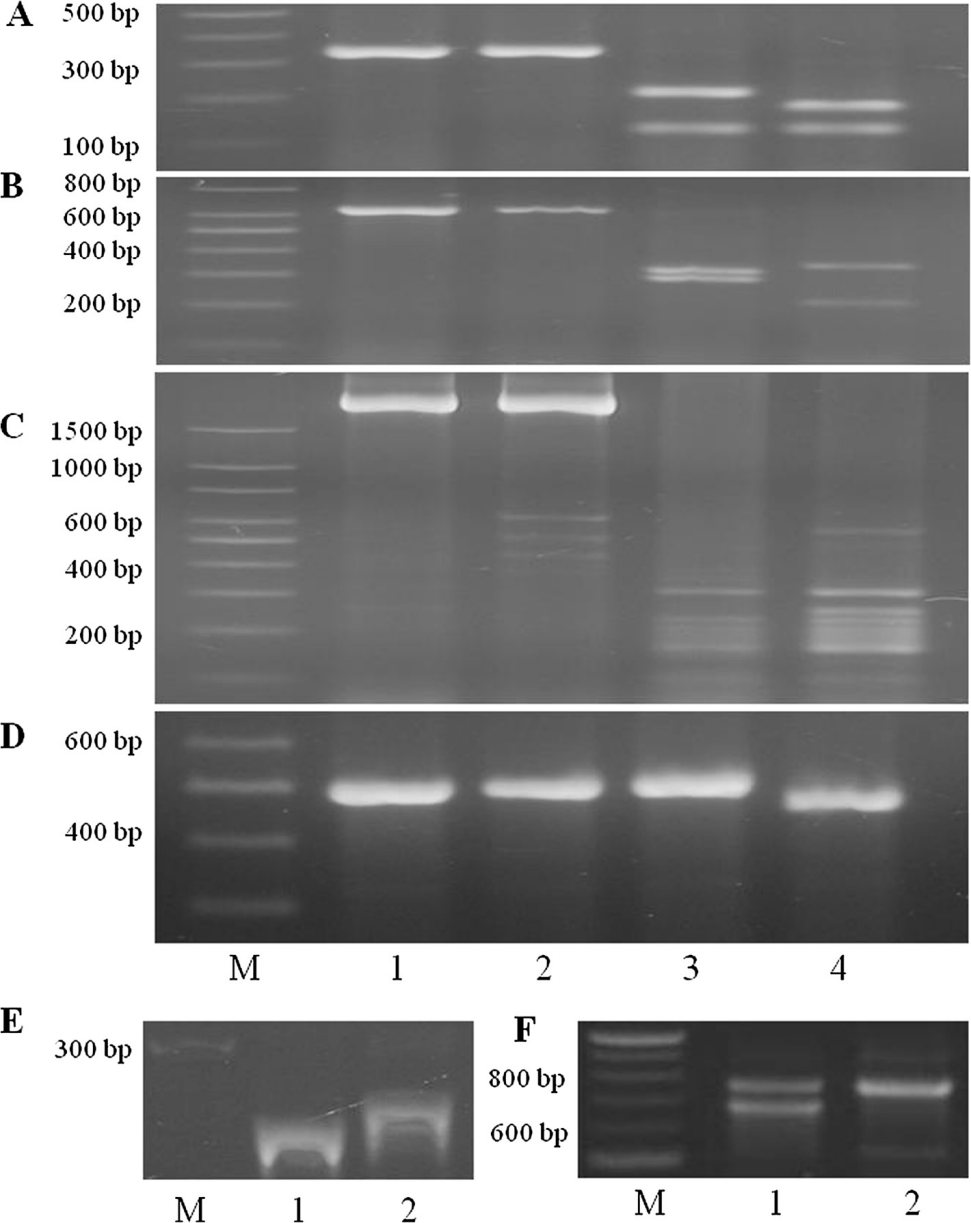
**PCR markers for six OPR and LOX genes, *TaOPR1-A, TaOPR2-A, TaLOX1-A, TaOPR7-B, TaLOX6-B, *and *TaLOX2-B*. A-F**) Lanes: 1 undigested Jagger 2 undigested 2174 3 digested Jagger 4 digested 2174. M Molecular marker. **A**) *TaOPR1-A* marker. Digestion with *Kpn*I shows polymorphic band patterns for Jagger (213 bp + 124 bp) and 2174 (36 bp + 177 bp + 124 bp) (the 36 bp band was run out of the gel). **B**) *TaOPR2-A* marker. Digestion with *Hinc*II shows polymorphic band patterns for Jagger (20 bp + 321 bp + 292 bp) and 2174 (342 bp + 215 bp + 77 bp) (the 20 bp and 77 bp bands were run out of the gel). **C**) *TaLOX1-A* marker. Digestion with *Scr*FI shows polymorphic band patterns for Jagger and 2174, in which 2174 has an extra ~250 bp fragment. **D**) *TaOPR7-B* marker. Digestion with *Dde*I shows polymorphic band patterns for Jagger (487 bp) and 2174 (35 bp + 452 bp) (the 35 bp band was run out of the gel). **E**) *TaLOX6-B* marker. PCR products show polymorphic band patterns for Jagger and 2174 with 8-bp indels. **F**) *TaLOX2-B* marker. PCR products show polymorphic band patterns for Jagger and 2174 with an extra lower band in Jagger. PCR products were separated in a 2% agarose gel (**A-D** and **F**) or 6% acrylamide gel (**E**).

A PCR marker, using primers OPRC1-ABD-F2 and OPRC1-R8, was developed for the *OPR* gene *TaOPR1-A*, the first *OPR* gene to be mapped on chromosome 1A of *T. aestivum*. The primers amplified a 337-bp fragment that was digested with *Kpn*I into 213 bp and 124 bp for the Jagger allele (GenBank: KF035075) and into 36 bp, 177 bp, and 124 bp for the 2174 allele (GenBank: KF035076) (Figure 4A). *TaOPR1-A* was mapped under the peak of *QHf.osu-1A* and 2.4 cM in genetic distance to *Xcfa2153* (Figure 2A) in the RIL population of Jagger × 2174.

The second *OPR* gene, *TaOPR2-A*, which showed 79% identity (Additional file 1) to *TaOPR1-A* within a range of 584 bp (E = 2e-112), showed complete linkage with a group of markers for *Yr17* translocated from chromosome 2N and under the peak of *QHf.osu-2A*. The wheat EST BE403717 that was mapped in 1AS-3 FL 0.86 deletion bin was used to blast against GenBank Nucleotide Collection (nr/nt) databases, and it showed 82% identity within a range of 318 bp (E = 1e-83) to an orthologous *OPR* gene in rice BAC from chromosome 6 (GenBank: AP004741). Specific primers were designed based on the grouped sequences retrieved from the wheat genomic DNA databases. The primers OPR22-C1-F3 and OPR-R2 amplified a 634 bp fragment that was digested with *Hinc*II into 20 bp, 321 bp and 292 bp for the Jagger allele (GenBank: KF035084) and into 342 bp, 215 bp, and 77 bp for the 2174 allele (GenBank: KF035085) (Figure 4B).

### Disassociations of *OPR* and *LOX* genes with mapped QTLs for Hessian fly resistance

**Figure 5:**
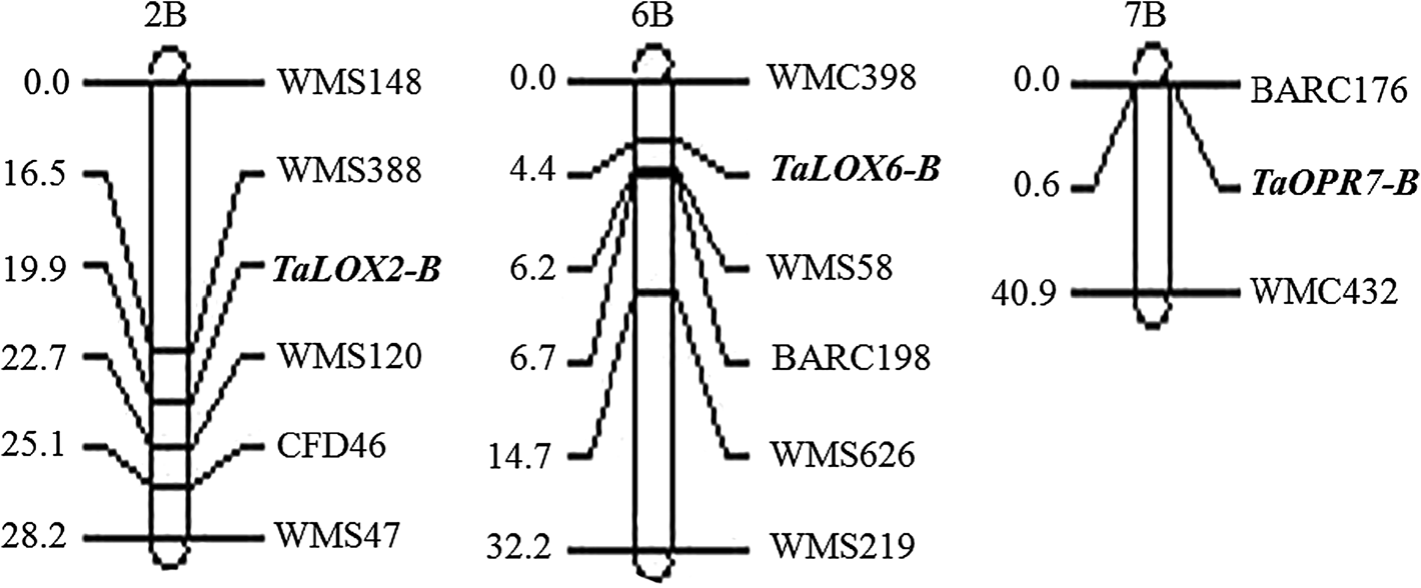
**Genetic maps of the *TaOPR7-B, TaLoX2-B***, **and *TaLOX6-B *genes. ***TaOPR7-B* was mapped on chromosome 7B (**A**), *TaLOX2-B* was mapped on chromosome 2B (**B**), and *TaLOX6-B* was mapped on chromosome 6B (**C**). Approximate distances in centi-Morgans (cM) and molecular markers are indicated on the left and the right, respectively.

In addition to the mapped associations of *TaOPR1-A* and *TaOPR2-A* with the two QTLs for Hessian fly resistance, another *OPR* gene *TaOPR7-B* was linked with *Xbarc176* on chromosome 7B (Figure 5A). Initially, an *OPR* gene present in rice BAC (GenBank: AP004707) was analyzed and used to search for the wheat orthologous *OPR* genes, because the rice BAC could be in a collinear region to wheat chromosome 1A. Specific forward primer OPR1-C1F1 was used to combine with conserved reverse primer OPR-R1 to amplify a single copy of the *OPR* gene mapped on chromosome 7B (*TaOPR7-B*). The chromosomal location of *TaOPR7-B* was confirmed by using CS nullisomic-tetrasomic lines (Figure 3B). The primers OPR-C1F1 and OPR-C1R1 amplified a 487 bp fragment that was digested with *Dde*I for the 2174 allele into 35 bp (GenBank: KF035087) and 452 bp but was not digested for the Jagger allele (GenBank: KF035086) (Figure 4D).

Three *LOX* genes were mapped in this study. Initially, a wheat EST (GenBank: BF482663) that was mapped in the 1AS-3 FL 0.86 deletion bin was used to blast against wheat gDNA databases (http://www.cerealsdb.uk.net) and GenBank EST databases for the homoeologous and paralogous *LOX* genes. This wheat EST was expected to reside in a region collinear with rice chromosome 5[45]. It showed 86% identity within a range of only 43 bp (E = 7e-05) to an orthologous gene in rice BAC from chromosome 5 (GenBank: AC136525), but it was more likely orthologous to other *LOX* genes in rice, with 87% identity in a range of only 294 bp (E = 5e-96) to one in chromosome 2 (GenBank: AP004184), with 72% identity (E = 2e-38) to two genes on chromosome 8 (GenBank: AP005816). Homoeologous chromosomes of hexaploid wheat could have three genes for each orthologous gene in rice. Primers were designed specifically to each of the groups retrieved from the sequences by BF482663.

The first *LOX* gene was mapped using primers LOX-C5-F5 and LOX-C5-R6 specific to chromosome 1A (Figure 3C). The primers amplified a fragment of approximately 2,500 bp that was digested with *ScrF*I into several fragments, distinguishable by an extra fragment of approximately 250 bp for the 2174 allele (GenBank: KF035089) compared with the Jagger allele (GenBank: KF035088) (Figure 4C). This *LOX* gene was mapped on chromosome 1A (*TaLOX1-A*) but outside of the *QHf.osu-1A* region (Figure 2A).

The second *LOX* gene was mapped using primers LOX0-F4 and LOX0-R6 that amplified a common band in both Jagger and 2174 and an additional lower band in Jagger (Figure 4F). PCR products from Jagger were purified from the agarose gel, and direct sequence analysis showed that it was part of a *LOX* gene (Additional file 2). The high quality sequence result indicated that it was from a pseudo gene in Jagger (GenBank: KF035090). The dominant marker for the *LOX* gene in Jagger was mapped with a linkage group of SSR markers on chromosome 2B (Figure 5B), and this gene was thus designed *TaLOX2-B*.

The third *LOX* gene was mapped using primers LOX-F4 and LOX-R6 that amplified PCR products of polymorphic size with approximately 264 bp in Jagger (GenBank: KF035091) and 272 bp in 2174 (GenBank: KF035092). The sequencing results confirmed that these PCR products were derived from part of a *LOX* gene, with an 8 bp deletion for the Jagger allele or an 8 bp insertion for the 2174 allele (Figure 4E). This *LOX* gene was mapped with a linkage group of SSR markers on chromosome 6B (Figure 5C), and this gene was thus designed *TaLOX6-B*.

**Figure 6:**
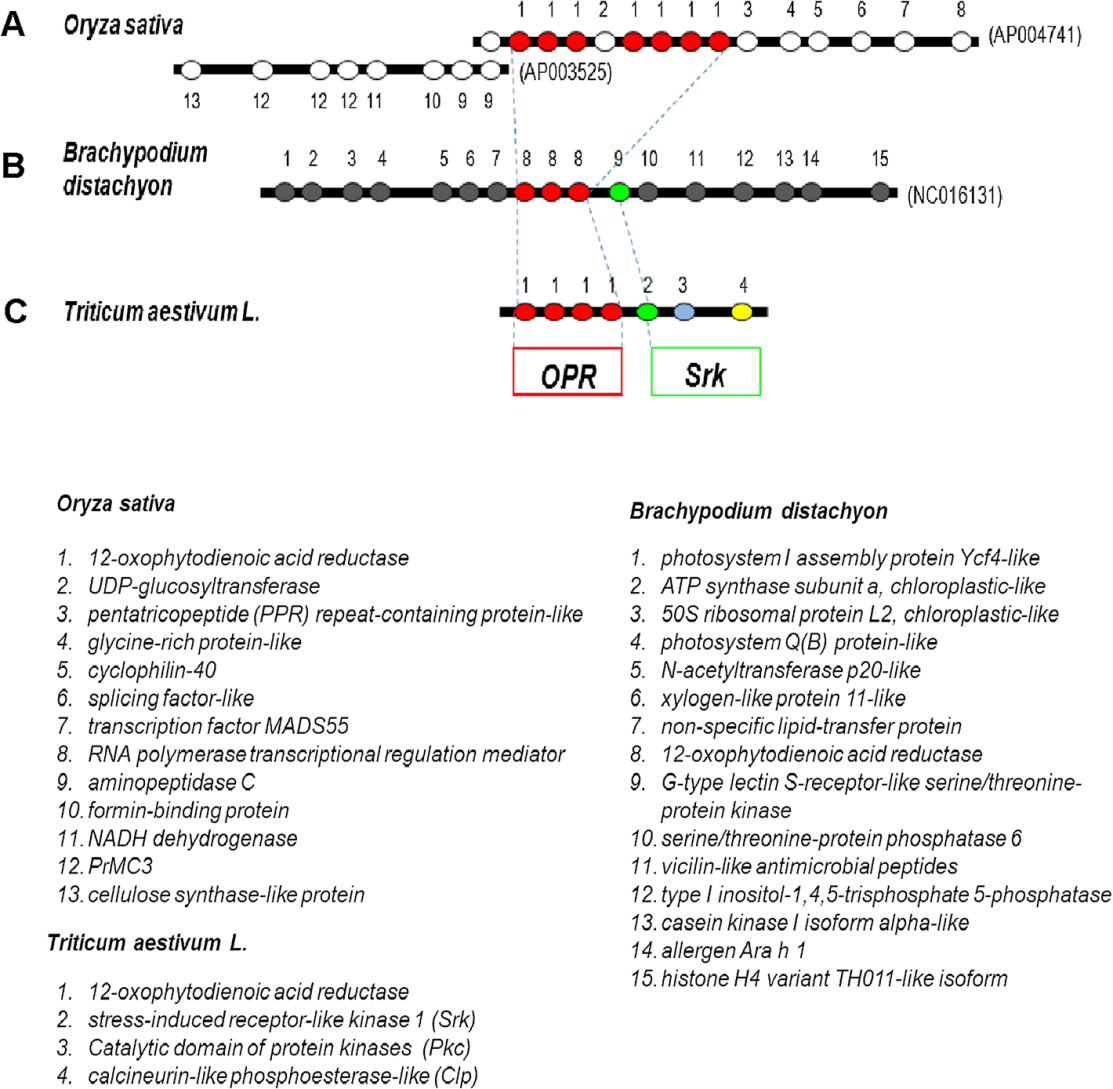
**Comparative map of the wheat OPR region on chromosome 1AS and in collinear regions from Brachypodium and rice.** The flanking genes for the OPR region in rice and Brachypodium are colored in white and grey, respectively. Flanking genes, *Srk, Pkc,* and *Clp* in wheat, are colored in green, blue, and yellow, respectively. Dotted lines indicate synteny of genes among wheat, rice, and Brachypodium.

### Fine collinearity at the *QHf.osu-1A* locus between wheat, rice and Brachypodium

*TaOPR1-A* was used to screen the *T. durum* BAC library, and one positive clone (73J24) was sequenced with low coverage to find genes that could be used to determine collinear regions of rice and Brachypodium. Four *OPR* genes (GenBank: KF035074, KF035077, KF035080, and KF035081), and three genes encoding stress-induced receptor-like kinase (*Srk*) (GenBank: KF035093), catalytic domain of protein kinases (*Pkc*) (GenBank: KF035099), and calcineurin-like phosphoesterase-like (*Clp*) (GenBank: KF035096) respectively, were present in the wheat BAC clone (Figure 6C). Allelic variation was observed in the *Srk* gene for the Jagger allele (GenBank: KF035094) and the 2174 allele (GenBank: KF035095), the *Clp* gene for the Jagger allele (GenBank: KF035097) and the 2174 allele (GenBank: KF035098), and the *Pkc* gene for the Jagger allele (GenBank: KF035100) and the 2174 allele (GenBank: KF035101). Howerver, no crossover was found between the *OPR* genes and these linked genes in the the *QHf.osu-1A* region.

*TaOPR1-A* displayed the highest identity to orthologous *OPR* genes in a rice BAC from chromosome 6 (GenBank: AP004741), which contains 7 *OPR* genes arranged in tandem (Figure 6A). Except for the wheat *OPR* genes, however, the other wheat genes (*Srk*, *Clp*, and *Pkc*) were not present in the flanking regions of the *OPR* genes in the two BAC clones shown in Figure 6A or in other BAC clones containing *OPRs* in rice. *TaOPR1-A* displayed the highest identity to three orthologous *OPR* genes in Brachypodium (GenBank: NC016131, Figure 6B), but again, except for the wheat *OPR* genes and *Srk* gene, the other wheat genes (*Clp* and *Pkc*) were not observed in the collinear region of Brachypodium. The orthologous genes in Brachypodium were not present in the flanking regions of the *OPR* genes in rice either.

## Discussion

A resistance gene against Hessian fly has been repeatedly mapped to the end of the short arm of chromosome 1A in previous studies, and it was suggested that this genomic region contained a cluster of major dominant resistance genes against multiple Hessian fly biotypes, including *H5*, *H9*, *H10*, *H11*, *H16*, *H17*, and *Hdic*[19,20,30,31]. However, all of the previous studies were performed in the tetraploid wheat *T. durum*. Our study is the first report that a resistance gene against Hessian fly exists in the short arm of chromosome 1A in bread wheat. The resistance gene at the *QHf.osu-1A* locus observed in hexaploid wheat cv. 2174 could be orthologous to one or more of the previously mapped resistance genes in tetraploid wheat. This study provides an effective resistance source to manage biotype GP that frequently damage bread wheat cultivars in the southern Great Plains. Further studies need to test if the *QHf.osu-1A* gene is allelic to genes *H16* and *H17* that confer resistance against Hessian fly biotype L, the most virulent and prevalent biotype in the eastern USA [31]. The resistance gene at *QHf.osu-1A* in 2174 and its derived cultivars can be immediately utilized to control Hessian fly biotype GP in winter wheat improvement programs in the southern Great Plains in USA. The gene may also be useful for pyramiding to prolong the effective periods of other resistance genes.

Mapping of *QHf.osu-1A* showed that it is closely linked with *Pm3a*. Previous studies indicated that *Pm3* was linked to *H9* at a genetic distance of 4.5 cM [61]. Several alleles have been characterized for the *Pm3* gene [63]. Leaf rust resistance gene *Lr10*, which has three paralogous genes and is effective against *Puccinia triticina* Eriks, was also mapped in the same chromosomal region on 1AS [64,65]. The presence of a resistance gene cluster in the gene-rich distal region of 1AS in bread wheat has made it difficult to determine which gene is the candidate for *QHf.osu-1A*. An effort was made to construct a fine physical map for *QHf.osu-1A*. However, low collinearity of the gene order in this region among wheat, rice, or Brachypodium indicates that the fine physical map for *QHf.osu-1A* cannot be established by using genome information from rice or Brachypodium only. The recent sequencing of wheat genomes may provide a powerful tool in cloning *QHf.osu-1A*.

Two *OPR* genes were identified in association with two QTLs for resistance against Hessian fly, but we cannot yet decisively conclude if either of the candidate *OPR* genes is responsible for *QHf.osu-1A*. The two QTLs were discovered in the same population, and resistance to Hessian fly is inseparable between the two QTLs. Two specific RIL lines (#23 and #26) have been selected to backcross with the 2174 parental line to generate progeny that will segregate for a single Hessian fly gene. In these backcross populations, *QHf.osu-1A* will occur in the heterozygous state, whereas *QHf.osu-2A* will be fixed in a homozygous genetic background. We expect that the two alleles of *QHf.osu-1A* will produce clear segregation for resistance to Hessian fly. BC_1_F_2-3_ populations will be used also to determine degree of dominance for the Hessian fly gene.

*TaLOX6-B* was mapped between *Xwmc398* and *Xbarc198* toward the centromere of wheat chromosome 6B. It was reported that *H25* was translocated from rye to the long arm of chromosome 6B of wheat [27], and thus, *TaLOX6-B* is not allelic to *H25*. *TaLOX2-B* is located between *Xwms388* and *Xwms120* on the long arm of chromosome 2B. *H20* was transferred from *T. durum* to chromosome 2B, and *H21* was translocated from rye to chromosome 2B in wheat. However, more information is needed to further clarify any allelic relationship of *TaLOX6-B* with either *H20* or *H21*. A recent study indicates that a novel wheat gene encoding a lectin-like protein (*Hfr-3*) was associated with response to Hessian fly [66]. The homoeologous *Hfr-3* genes are located on group 7 chromosomes of the hexaploid wheat. Yet, no Hessian fly resistance gene was reported on chromosome 7B where *TaOPR7-B* was mapped.

## Conclusions

In summary, a frequent objective of wheat breeding is to pyramid multiple genes for resistance to diseases and insects into a single cultivar. Compared with 2174, Jagger attained a translocated fragment that contains *Lr37*, *Yr17* and *Sr38* conferring resistance to leaf rust, stripe rust, and stem rust, but Jagger lost the allelic region containing the resistance gene on *QHf.osu-2A* against Hessian fly. The molecular marker can greatly facilitate the identification of such a candidate gene for the *QHf.osu-1A* locus, enabling opportunities for functional analysis which can then be used to better understand the defense mechanisms of wheat plants in response to this major insect.

## Methods

### Hessian fly populations

A Kansas population of Hessian fly consisting of predominantly biotype GP [67] was used in this study. The population was maintained on Hessian fly susceptible wheat seedlings (‘Karl 92’) in the greenhouse. Karl 92 was used as a susceptible control and WGRC42 was used as a resistant control to test the Hessian fly population [19].

### Plant materials and DNA isolation

Based on observations on seedling plants, Jagger is susceptible to Hessian fly biotype GP infestation whereas 2174 is resistant; therefore, the Jagger × 2174 population of 154 F_6:8_ recombinant inbred lines (RILs) were used to map genes associated with resistance to Hessian fly. Wheat genomic DNA was extracted from leaf tissue of each RIL plant as described previously [68].

### Evaluation of Hessian fly resistance

Parental lines and 154 F_6:8_ RILs were evaluated for phenotypic reaction to Hessian fly infestation in growth chambers at 18 ± 1°C with a 14 h:10 h (light: dark) photoperiod as described previously [20,34,69]. Briefly, seedling plants were infested with mated Hessian fly females. Three weeks after infestation, seedling plants were examined to identify resistant and susceptible phenotypes. Susceptible plants were stunted, dark green, and harbored live larvae, whereas resistant plants grew normally with light green color and dead larvae [67]. Hessian fly reactions were recorded and a random subset of the RILs was confirmed with additional replications (three totally).

Resistance screening was carried out in greenhouse as described previously [19]. Specifically, approximately 20 seeds of each wheat line were planted in a flat. A flat with soil was divided into 22 rows with a line divider, and each row was further divided into two half rows. Therefore in each flat, a total of 44 wheat lines could be planted. Along with wheat lines from a mapping population, two susceptible control wheat lines, Karl 92 and Jagger (the susceptible parent), and two resistant control wheat lines, 2174 (putative resistant donor) and WGRC42 (containing the R gene *Hdic*), were also planted in each flat. Wheat plants were infested at one leaf-stage (the second leaf just emerged) with Hessian fly adults under a cheese cloth tent. Females deposit eggs on the adaxial surface of the first leaf. Infestation was stopped when the egg density reaches 8 per leaf, which usually results in ~5 larvae per plant. Resistant and susceptible plants were phenotyped three weeks after infestation. Phenotypes of resistant and susceptible plants were distinct and easy to score. Resistant plants grew normally with light green color and susceptible plants were stunt with dark-green color. Plants with a resistant phenotype were further dissected to check for dead larvae. Plants without dead larvae were taken as escapes and were excluded from the data set.

### QTL analysis

A total of 404 SSR markers were assembled in linkage groups using MapMaker 3.0 program, and a QTL was identified using the WinQTLCart 2.5 program as previously described [37,70]. Other unlinked markers were tested using correlation analysis to examine which markers might be related to Hessian fly resistance using SAS software (SAS 9.1, SAS Institute Inc. Cary, NC, USA).

### Gene-specific marker development

Wheat ESTs encoding LOX (GenBank: BE403717) and OPR (GenBank: BE482663) were found present in the deletion bin (1AS-3 FL 0.86) on chromosome 1A, and these ESTs were used to develop markers for mapping. The genomic sequences of the orthologous *OPR* and *LOX* genes in rice are available in GenBank databases, including *OPR* genes in rice BAC from chromosome 1 (GenBank: AK100034), chromosome 6 (GenBank: AP004741), and chromosome 8 (GenBank: AP004707); and *LOX* genes from chromosome 2 (GenBank: AP004184), chromosome 5 (GenBank: AC136525), and chromosome 8 (GenBank: AP005816). Rice BACs and wheat EST sequences were used to blast against wheat gDNA databases (http://www.cerealsdb.uk.net) to retrieve homoeologous and paralogous sequences for each *TaOPR* and *TaLOX* genes. Multiple sequence alignments showed that the homoeologous and paralogous *TaOPR* and *TaLOX* genes are variable, which made it possible to design genome-specific primers for each gene. Gene markers developed were used to analyze the RIL population and evaluated for linkage (Table 1).

**Table 1:** **Primers used for detecting allelic variation in *TaOPR1-A, TaOPR2-A, TaOPR7-B, TaLOX1-A, TaLOX2-B, TaSrk, TaClp*, and *TaPkc *in hexaploid wheat**

**Primer**	**Sequence (5′-3′)**	**Loci**	**PCR size (bp)**	***T***_***m ***_**(°C)**	**Restriction site**
OPRC1-ABD-F2	CCGTCGACGCCGGTACG	*TaOPR1-A*	337	62	*Kpnl*
OPRC1-R8	GGCCGCCGATCTCCCT	*TaOPR1-A*			
OPR22-C1-F3	TCTGCTTTCCTCTGCTCGTC	*TaOPR2-A*	634	55	*Hincll*
OPR-R2	TTCATGGTTCAATGACACATCAAGG	*TaOPR2-A*			
OP1-C1F1	AAAAGGTTTTCACCTCGATGATCGGGG	*TaOPR7-B*	487	55	*Ddel*
OP1-R1	CCGATGGCCCTGCACCTCGTCATCG	*TaOPR7-B*			
LOX-C5-F5	CATCCTGAATAAAGAACCTC	*TaLOX1-A*	2500a	55	*ScrFl*
LOX-C5-R6	GATCATATGGAGACGCTGTT	*TaLOX1-A*			
LOX0-F4	GGAGCACGGCCTCAAGCTC	*TaLOX2-B*	680a	52b	dominant band
LOX0-R6	CATGTGGTTTATTTTAGCTCTGTAGA	*TaLOX2-B*			
LOX-F4	CTGCGTCGAGCCCTACATCATCG	*TaLOX6-B*	264/272	62	8-bp indels
LOX-R6	GGGCCAGCCCCCGGCTGACG	*TaLOX6-B*			
Srk-F3	CGGACCACTTCAACCATAGG	*TaSrk*	1437	56	
Srk-R2	ACCTTTCACAGTCTGCAATGGCAATGCT	*TaSrk*			
CLP-F1	TCCATCTCCTATGGCTTCTT	*TaClp*	1648	55	*Rsal*
CLP-R2	GGTGGGGGTTGCCTATCCAG	*TaClp*			
Pkc-F3	GAGAGAAGCAATTCAGGGCT	*TaPkc*	911	55	
Pkc-R2	CCAGATTACAATTTTAAAGAGAAG	*TaPkc*			

^a^ Estimated product size.

^b^ A touch-down program (60°C down to 52°C for annealing temperature) was performed before the regular was performed (see details in Methods).

### Polymerase chain reaction (PCR)

All PCR reactions were performed in a 25 μl reaction under the following conditions: 1 denaturation cycle at 94°C for 5 min; 40 cycles of 94°C for 30 s, 52-62°C (based on primer annealing temperature) for 30 s, and 72°C for 30 s – 2.5 min (based on fragment length), then a final extension step at 72°C for 10 min before cooling to 4°C. PCR amplified fragments were separated on 2% agarose gels through electrophoresis in 1× TAE buffer or 6% acrylamide gel in 0.5× TBE buffer. DNA banding patterns were visualized under UV light with ethidium bromide staining.

## Abbreviations

OPR: 12-oxo-phytodienoic acid reductase; LOX: Lipoxygenase; RILs: Recombinant inbred lines; QTL: Quantitative trait loci; Hf: Hessian fly; SSR: Simple sequence repeat; JA: Jasmonic acid; OPDA: 12-oxo-phytodienoic acid; AOS: Allene oxide synthase; OPR3: 12-oxo-phytodienoic acid reductase 3; GP: Great plains; EST: Expressed sequence tag; BAC: Bacterial artificial chromosome; gDNA: Genomic DNA; Srk: Induced receptor-like kinase; Pkc: Catalytic domain of protein kinases; Clp: Calcineurin-like phosphoesterase-like.

## Competing interest

The authors declare that they have no competing interests.

## Authors’ contributions

CTT performed the experiments and analyzed data. BFC provided plant materials for the project and helped edit the manuscript. MSC performed phenotype experiments for Hessian fly resistance. YQG performed analysis of comparative genomics and provided BAC clones. CTT and LY designed the project and wrote the manuscript. All authors read and approved the manuscript.

## Supplementary Material

Additional file 1 **Sequence comparison of *TaOPR1-A *and *TaOPR2-A.*** Click here for file

Additional file 2 **Sequence comparison of a *LOX *gene between wheat and rice.** Click here for file
