# Supplementary material for: Genetic association of OPR genes with resistance to Hessian fly in hexaploid wheat
Source: BMC Genomics. 2013 Jun 1;14:369. doi: 10.1186/1471-2164-14-369 (PMC3674912; doi:10.1186/1471-2164-14-369)
Supplement: Additional file 1 — Sequence comparison of TaOPR1-A and TaOPR2-A. [file 1471-2164-14-369-S1.doc]

**Supplementary Figure 1**. **Sequence comparison of *TaOPR1-A* and *TaOPR2-A***. Sequences were obtained from the 3’end regions of the *OPR* genes. Polymorphic sites were highlighted in grey.
