# Supplementary material for: Genetic association of OPR genes with resistance to Hessian fly in hexaploid wheat
Source: BMC Genomics. 2013 Jun 1;14:369. doi: 10.1186/1471-2164-14-369 (PMC3674912; doi:10.1186/1471-2164-14-369)
Supplement: Additional file 2 — Sequence comparison of a LOX gene between wheat and rice. [file 1471-2164-14-369-S2.doc]

**Supplementary Figure 2. Sequence comparison of the *LOX2-B* gene between wheat and rice**. The wheat *LOX2-B* gene was obtained from cv. Jagger using primers LOX0-F4 and LOX0-R6. The rice orthologue of the *LOX2-B* gene is *lipoxygenase-2* gene (GenBank accession# EU085425).
